# PSA density of the lesion: a mathematical formula that uses clinical and pathological data to predict biochemical recurrence in prostate cancer patients

**DOI:** 10.1590/0100-6991e-20212965

**Published:** 2021-09-28

**Authors:** PEDRO HENRIQUE REZENDE JUNQUEIRA, GABRIEL ARANTES DOS SANTOS, MARCELO XAVIER, POLIANA ROMÃO, SABRINA REIS, MIGUEL SROUGI, WILLIAN CARLOS NAHAS, CARLO CARMARGO PASSEROTTI

**Affiliations:** 1 - Hospital Oswaldo Cruz, Centro de Cirurgia Robótica - São Paulo - SP - Brasil; 2 - Faculdade de Medicina da Universidade de São Paulo, Urologia, Laboratório de Investigação Médica - São Paulo - SP - Brasil; 3 - Instituto do Câncer de São Paulo, Urologia - São Paulo - SP - Brasil

**Keywords:** Recurrence, Prognosis, Prostate-Specific Antigen, Prostatic Neoplasms, Recidiva, Prognóstico, Neoplasias da Próstata, Antígeno Prostático Específico

## Abstract

A main challenge in the clinical management of prostate cancer is to identify which tumor is aggressive and needs invasive treatment. Thus, being able to predict which cancer will progress to biochemical recurrence is a great strategy to stratify prostate cancer patients. With that in mind, we created a mathematical formula that takes into account the patients clinical and pathological data resulting in a quantitative variable, called PSA density of the lesion, which has the potential to predict biochemical recurrence. To test if our variable is able to predict biochemical recurrence, we use a cohort of 219 prostate cancer patients, associating our new variable and classic parameters of prostate cancer with biochemical recurrence. Total PSA, lesion weight, volume and classic PSA density were positively associated with biochemical recurrence (p<0.05). ISUP score was also associated with biochemical recurrence in both biopsy and surgical specimen (p<0.001). The increase of PSA density of the lesion was significantly associated with the biochemical recurrence (p=0.03). Variables derived from the formula, PSA 15% and PSA 15^2^, were also positive associated with the biochemical recurrence (p=0.01 and p=0.002 respectively). Logistic regression analysis shows that classic PSA density, PSA density of the lesion and total PSA, together, can explain up to 13% of cases of biochemical recurrence. PSA density of the lesion alone would have the ability to explain up to 7% of cases of biochemical recurrence. In conclusion, this new mathematical approach could be a useful tool to predict disease recurrence in prostate cancer.

## RESUMO

Um dos principais desafios no manejo clínico do câncer de próstata é identificar qual tumor é agressivo e precisa de tratamento invasivo. Assim, ser capaz de prever qual irá progredir para recorrência bioquímica é uma ótima estratégia para estratificar pacientes com câncer de próstata. Pensando nisso, criamos uma fórmula matemática que leva em consideração os dados clínicos e patológicos resultando em uma variável quantitativa, denominada densidade de PSA da lesão, que tem potencial para predizer recidiva bioquímica. Para testar se nossa variável é capaz de predizer recorrência bioquímica, usamos uma coorte de 219 pacientes com câncer de próstata, associando nossas variáveis e parâmetros clássicos como a recorrência bioquímica. PSA total, peso da lesão, volume e densidade de PSA clássico foram associados com recorrência bioquímica (p<0,05). O escore ISUP também foi associado à recorrência bioquímica na biópsia e na amostra cirúrgica (p<0,001). O aumento da densidade do PSA da lesão foi significativamente associado à recidiva bioquímica (p=0,03). As variáveis ??derivadas da fórmula, PSA 15% e PSA 15^2^, também foram associadas positivamente à recorrência bioquímica (p=0,01 e p=0,002 respectivamente). A análise de regressão logística mostra que a densidade do PSA clássico, do PSA da lesão e PSA total, juntos, podem explicar até 13% dos casos de recorrência. A densidade de PSA da lesão por si só poderia explicar até 7% dos casos de recorrência. Em conclusão, esta nova abordagem matemática pode ser uma ferramenta útil para prever a recorrência da doença no câncer de próstata.

## INTRODUCTION

Prostate cancer (PCa) is the second most common neoplasm and the fifth leading cause of cancer death in men^1^. Furthermore, predictions indicate that the number of PCa cases has been increasing and, together with this increase in incidence, the number of operations to treat the disease has increased considerably^2^ ^-^ ^4^. The widespread use of prostate specific antigen (PSA) has increased the detection rates of this cancer at earlier stages. However, up to 20% of clinically localized cases may present disease recurrence after local radical treatment in a 10 year follow-up period^5^.

This phenomenon occurs because current clinical and pathological parameters fail to determine accurate prognosis in many cases^6^. In this regard, many efforts are concentrated on finding predictors of biochemical recurrence (i.e. the increase of serum PSA after treatment). 

In the current study, we evaluate whether the present surgical specimen information can be a predictor of biochemical recurrence. Thus, we have created a formula to assess the PSA density of the lesion, trying to offer a way to include some data from patients, which have been neglected by current methods that can predict biochemical recurrence.

## METHODS

### Experimental Design

This is a retrospective longitudinal analytical study that was carried out after approval by the Ethics and Research Committee of the Hospital das Clínicas of the University of São Paulo (under the number: 1.955.609).

Initially, 372 male patients, aged between 43 and 81 years, from a private clinic of a single surgeon (CCP), operated between September 2009 and June 2019 were evaluated. However, 76 patients were excluded because they were regularly followed up according to the protocol. Another 64 patients were excluded because they did not present an exact description of the weight of the lesion or the weight of the prostate in the pathological exam. Thirteen patients were excluded for having hormonal block prior to surgery. Thus, the population of the present study was composed of 219 patients.

The PSA density of the lesion of all the patients was calculated and at the end, this calculation was correlated with the presence of tumor recurrence. We have considered biochemical recurrence the finding of PSA values greater than 0.2ng/dL in two consecutive postoperative samples. The first postoperative PSA measurement occurred eight weeks after the operation. Patients with values greater than or equal to 0.2ng/dL underwent a new confirmatory dosage^7^. 

### Evaluation of the PSA density of the lesion

To analyze the density of the PSA we used anatomopathology information. We estimated the weight of the primary neoplastic lesion as well as the multicentric lesions using the method standardized by the College of American Pathologists. This method defines that the specimen from radical prostatectomy can have its neoplasia percentage quantified by: careful visualization, being complemented by objective data such as measures of tumor dimensions and; the number of blocks involved by the tumor in relation to the number of total blocks. It is important to highlight that the pathology analysis of the samples was not necessarily performed by the same pathologist^8^ ^,^ ^9^.

We calculated the difference between the weight of the prostate (WP) and the weight of the neoplastic lesion (WL

Firstly, we calculated the weight of the non-neoplastic (benign) portion of the prostate (BP) with the data obtained from the complete anatomopathology examination of each patient (which systematically reports the prostatic weight and the estimated percentage of the prostatic volume represented by tumor tissue). Differences between the weight of the prostate (WP) and the weight of the neoplastic lesion (WN), were assessed according to the following formula: BP = WP - WN.

Then, we performed the calculation of the PSA produced only by the benign portion of the prostate (PSABP). We considered the PSA density of 15% as the cutoff to estimate PSA production by the non-neoplastic portion of the prostate. This value of 0.15 is based on the cutoff of the predictor called classic prostate PSA density, traditionally used as a predictor for patient selection for prostate biopsy and as a parameter for the management of patients under active surveillance. This value is obtained by dividing the total PSA value and the prostate volume, then the following formula was used: PSABP = 0.15 x BP^3^ ^-^ ^5^. Subsequently, we calculated the PSA produced by the neoplastic portion (PSA of the lesion), using the formula: PSA of the lesion = total PSA - PSABP.

After calculating the PSA of the lesion, a correction factor was added, since there is a possibility that the PSA calculation value of the lesion is negative. Therefore, this value was squared in order to correct this lesion, generating a PSA value - fifteen squared (PSA15²): PSA15² = (PSA of the lesion)². Lastly, the PSA density of the neoplastic lesion was calculated (DNL) by the division between PSA15² and WL (DNL = PSA15²/WN). 

The steps can be simplified by performing the calculation using the formula:

PSAdensityofthelesion(DNL)=[TotalPSA−0.15(WP–WL)]²/WL.

### Application of the formula

**Figure 1:**
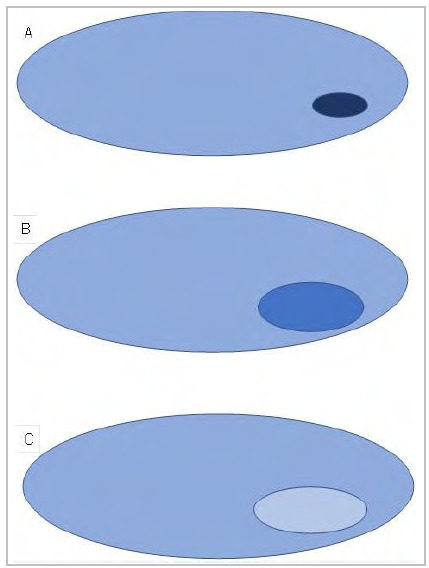
Illustration of the prostate models. The larger ellipse represents the entire prostate and the smaller ellipse represents the neoplastic lesion. The blue tone estimates the increase of produced PSA (the lighter, the lower the PSA production) by the fabric in question. A) Small lesion with high DNL. B) Larger lesion with low DNL. C) Lesion with very low DNL.

Therefore, we propose a way to include patient profiles that are commonly neglected by current methods of relapse predictors. For the best elucidation of how these data are used, we demonstrate three models of prostate, represented with data from patients who participated in our study:

Example 1: patient with total PSA of 4.4ng/dL, prostate volume of 57.6cm^3^. The anatomopathology evidence showed that the prostate weight was 50 grams and 5% of the prostate gland was occupied by the neoplasm (i.e.: lesion weight: 2.5g), with a classic PSA density of 0.08 (considered low, predictor of low aggressiveness) and DNL of 2.96 (new parameter, considered high, bearing a possible predictor of aggressiveness). The patient in question evolved with biochemical recurrence. It is a patient model with a potential small aggressive lesion that could induce the production of a large amount of PSA per gram of injury. Therefore, this is a profile of a patient with a greater possibility of biochemical recurrence, susceptible to being neglected by current predictors (Figure 1A).

Detailed calculations of example 1:

**Figure:**
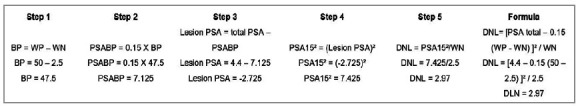

Example 2: patient with total PSA of 6.0ng/dL, prostate with a volume of 34cm^3^, prostate weight of 35g and 10% of the prostate volume occupied by the neoplasm, classic PSA density of 0.17 (considered high, predictor of aggressiveness) and DNL of 0.46 (new parameter, considered low, with a possible predictor of little aggressiveness). The patient in question did not present biochemical recurrence. It is a patient model that presents a potential little aggressive lesion that could induce the production of a small amount of PSA per gram of lesion. Therefore, this is a profile of a patient with less chance of biochemical recurrence, susceptible to being treated with unnecessarily adjuvant therapy, based on currently available predictors (Figure 1B).

Example 3: patient with total PSA of 3.1ng/ dL, prostate with a volume of 40cm^3^, prostate weight of 40g, 10% of the prostate volume occupied by the neoplasia, classic PSA density of 0.07 (considered low, predictor of low aggressiveness) and DNL of 1.32 (new parameter, considered high, bearing a possible predictor of aggressiveness). The patient in question evolved with biochemical recurrence. This is a patient model with a likely aggressive injury that could cause little or no increase in PSA levels per gram of injury. Therefore, this is a profile of a patient with a greater possibility of biochemical recurrence, susceptible to being neglected by current predictors (Figure 1C).

The study was approved by the local ethics committee under the number 64119817.6.0000.0065. 

### Statistical analysis

We calculated a post hoc sample power, using the G Power 3.1 program based the determination coefficient (r^2^) obtained from the models generated from the multiple Logistic Regression. We considered the sampling error of 5% and a 95% confidence interval, and the minimum significant sample for the study was 68 patients.

The collected data were initially plotted on a spreadsheet using the Microsoft Excel software (2013) and later analyzed with the aid of the SPSS software (23.0). The characterization of the patient’s profile was performed by absolute (n) and relative (%) frequency for categorical variables. Mean, standard deviation, median, minimum, maximum and interquartile range for continuous variables were considered. In this study, the normality was tested by the Shapiro-Wilk test. The comparison of the postoperative outcomes with categorical exploratory variables was performed using the Pearson chi-square and Post hoc chi-square test. The agreement between ISUP in surgical specimen and ISUP in biopsies was made using the Kappa test (data no shown). Regarding continuous variables, analyzes were performed using Student’s t and Mann-Whitney tests. In order to explore the contribution of continuous and categorical exploratory variables under the postoperative outcome, multiple logistic regression was performed using the conditional step-forward method. In all analyzes, the level of significance adopted was 5% (p<0.05).

## RESULTS

The patients’ clinical variables are shown in Tables 1 and 2. The average age was 62.51± 7.60 years. We observed a biochemical recurrence rate of 18.3%. 

**Table 1:** Characterization of continuous exploratory variables.

	Mean ± Standart Deviation	Median	Interquartile range
Age	25p - 75p	62.00	58.00 - 68.00
Total PSA	6.48 ± 4.84	5.10	4.00 - 7.50
PSA of the lesion	0.83 ± 5.10	0.13	1.68 - 2.02
PSA of the lesion 15²	26.54 ± 159.14	3.31	0.79 - 12.15
PSA density of the lesion	2.70 ± 7.49	0.60	0.15 - 2.01
Weight of the prostate	45.15 ± 17.84	44.00	34.00 - 55.00
Prostate Volume	44.94 ± 19.91	41.60	31.50 - 54.30
Lesion Volume (%)	17.66 ± 12.30	15.00	10.00 - 24.00
Lesion Weight	7.49 ± 5.46	6.00	3.50 - 10.00
Classic PSA Density	0.16 ± 0.11	0.13	0.09 - 0.17

**Table 2:** Characterization of categorical exploratory variables.

	n	%
Biochemical Recurrence		
No	179	81.7
Yes	40	18.3
Biopsy ISUP		
1	74	33.8
2	70	32.0
3	37	16.9
4	29	13.2
5	9	4.1
Surgical Specimen ISUP		
1	21	9.6
2	104	47.5
3	61	27.9
4	11	5.0
5	22	10.0
Extraprostatic Extension		
No	133	60.7
Yes	86	39.3
Margins		
Negative	156	71.2
Positive	63	28.8
Potency		
Impotent	57	26.0
Potent	162	74.0
Continence		
Continent	211	96.3
Incontinent	8	3.7

n: Absolute Frequency; %: Relative Frequency.

Table 3 depicts the data regarding the comparison between patients with or without biochemical recurrence. The median age of patients with biochemical recurrence was 61.5 years and of those without biochemical recurrence was 63.0 years (p=0.18). Prostate weight and volume was not associated with biochemical recurrence (p=0.47 and p= 0.56 respectively). 

**Table 3:** Result of biochemical recurrence comparison with continuous exploratory variables.

	Biochemical Recurrence		
	No	Yes	p*
	Median (25p - 75p)	Median (25p - 75p)	
Age	63.00 (59.00 - 68.00)	61.50 (55.25 - 65.75)	0.18
Total PSA	5.00 (3.98 - 7.10)	6.29 (4.40 - 11.26)	0.01
PSA of the lesion 15%	-0.12 (-1.83 - 1.76)	1.10 (-0.63 - 5.88)	0.002
PSA of the lesion 15²	3.20 (0.77 - 11.22)	4.53 (0.81 - 34.60)	0.01
PSA density of the lesion	0.54 (0.15 - 1.88)	0.68 (0.17 - 4.03)	0.03
Prostate Weight	45.00 (32.00 - 55.00)	40.00 (35.00 - 50.00)	0.47
Prostate Volume	41.60 (32.00 - 54.30)	41.00 (29.25 - 54.00)	0.56
Lesion Volume (%)	15.00 (10.00 - 20.00)	20.00 (10.25 - 30.00)	0.02
Lesion Weight	6.00 (3.47 - 9.00)	7.75 (4.68 - 12.00)	0.03
Classic PSA Density	0.12 (0.09 - 0.17)	0.16 (0.12 - 0.26)	0.005

* Mann-Whitney test.

The total PSA values were different between patients who had recurrence or not (6.29 vs 5.00 respectively, p=0.01). The increase of lesion and weight volume was associated with biochemical recurrence (p=0.02 and p=0.03 respectively). The Classic PSA density was also positively associated with biochemical recurrence (p=0.005)

The new developed PSA density of the lesion was assessed, and we found that the increase of this variable was significantly correlated with the biochemical recurrence (p=0.03). PSA 15% and PSA 15^2^ were also positively associated with the biochemical recurrence (p=0.01 and p=0.002 respectively)

**Figure 2:**
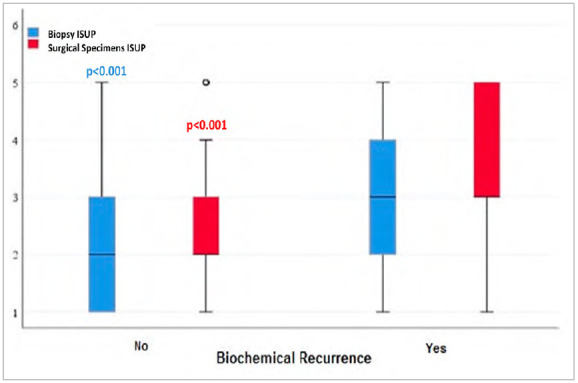
Comparison ISUP score between biopsy and surgical specimens in patients with or without biochemical recurrence. *Mann-Whitney test.

Additionally, the ISUP score between the group with or without biochemical recurrence (Figure 2) was evaluated, and the increase in the ISUP score was associated with the biochemical recurrence in both biopsy and surgical specimens of PC samples (p<0.001).

Finally, the multiple logistic regression (Table 4) indicated that the classic variables as PSA density, PSA density of the lesion and total PSA, together, can explain up to 13% of cases of biochemical recurrence. PSA density of the lesion, according to this model, would have the ability to explain up to 7% of cases of biochemical recurrence.

**Table 4:** Result of multiple logistic regression models using the Backward-LR method.

Model	Predictors	r^2^	B	Standard Error	Wald	p
1	Total PSA	0.15	0.13	0.07	3.88	0.05
	PSA of the lesion 15²		0.00	0.01	0.03	0.86
	PSA density of the lesion		-0.10	0.08	1.56	0.21
	Lesion Weight		0.05	0.04	1.63	0.20
	Classic PSA Density		4.47	2.84	2.48	0.11
2	Total PSA	0.15	0.14	0.07	4.17	0.04
	PSA Density of the lesion		-0.09	0.04	3.85	0.04
	Lesion Weight		0.05	0.03	2.07	0.15
	Classic PSA Density		4.47	2.82	2.51	0.11
3	Total PSA	0.13	0.16	0.06	6.22	0.01
	PSA Density of the lesion		-0.09	0.04	4.54	0.03
	Classic PSA Density		4.23	2.79	2.30	0.13
4	Total PSA	0.12	0.20	0.06	12.78	<0.01
	PSA Density of the lesion		-0.07	0.04	3.69	0.04
5	Total PSA	0.05	0.13	0.04	9.22	<0.01

## DISCUSSION

The curative treatment for PCa has highly been carried out in recent decades^10^. Robotic radical prostatectomy is certainly one of the greatest advances. However, treatment is not free from adverse effects and there is always the possibility of an unfavorable outcome despite the use of the best available methods. Overtreatment is a real problem for PCa, thus being able to predict those patients who need invasive treatment, and those who have an indolent disease is highly necessary^11^. In addition, the indiscriminate use of PSA levels has been associated with a high rate of overdiagnosis and excessive treatment^12^ ^-^ ^14^.

Normally, after treatment, the PSA levels of PCa patients drop to zero. Biochemical recurrence is a phenomenon in which the PSA increases again, indicating the disease recurrence^15^. Therefore, being able to predict biochemical recurrence is a great strategy to early identify aggressive tumors. 

In this study, we present the PSA Density of the lesion, a new parameter that is intended to help predict biochemical recurrence. Other authors have tried to use derivations of PSA density^6^; however, no studies were found that used the same criteria used in this protocol. Therefore, it is an original parameter. It is derived from a calculation that takes into account the total PSA, the weight of the prostate and the weight of the neoplastic lesion. We also tested other new parameters such as and PSA of the lesion 15% and PSA of the lesion15², used in the calculation of the Lesion Density. Other already established parameters, such as total PSA, classic density of PSA, fraction of the prostate affected by the tumor, pathology signs of extra-prostatic extension, ISUP graduation, prostate weight, prostate volume and lesion weight were used.

DNL is an estimate of the increase in PSA concentration caused by the gram of the neoplastic lesion. The other existing derivative methods of PSA do not take into account their particular specific correlation with the weight of the neoplastic lesion. Therefore, it is an attempt to consider that there may be small lesions, but with the capacity to cause a relative high production of PSA or even large neoplastic lesions that do not significantly increase PSA. These would be, at first, less aggressive, while those would have greater power to cause a clinically unfavorable evolution.

It is also proposed, with this calculation model, to correct part of the distortions that occurs, for example, in patients with poorly differentiated lesions and insignificant PSA production, which would lead patients with this profile to have their disease underestimated.

The presented results reveal that the proposed new parameter has a statistically significant correlation with the outcome, both in univariate analysis and in multiple logistic regression. The clinical applicability of this new parameter would be for the selection of patients with a higher risk of biochemical recurrence. For these patients, adjuvant treatments would be considered more seriously instead of waiting for the patient to present biochemical recurrence to administer rescue therapies. 

The pathology graduation according to the ISUP criteria showed a positive association when the outcome of biochemical recurrence was considered. Other authors have also identified the pathology graduation as a predictor of biochemical recurrence^16^. In a recent study, the rate of biochemical recurrence in 1,754 men who underwent radical prostatectomy and concluded that ISUP score was assessed as the most important predictor for biochemical recurrence in high-risk patients^15^. Our data are in agreement with the literature in this topic.

The total PSA value was confirmed as an independent predictor of biochemical recurrence, both in the univariate analysis and in multiple logistic regression, which is widely supported by the literature^17^ ^-^ ^19^. The classic density of PSA also showed a statistically significant correlation and this is in agreement with what we found in our literature review^17^ ^,^ ^18^. In a series of 784 patients undergoing robotic radical prostatectomy, it was identified that the density of PSA is an independent predictor of biochemical recurrence in patients undergoing treatment with a curative purpose^20^.

We analyzed the potential correlation of the prostate weight provided by the pathology examination and did not find any significant association. When we considered the prostate volume measured by preoperative ultrassonograpy and tried to correlate it with the outcome of biochemical recurrence, we did not find any statistically significant values. In contrast, other authors have already demonstrated the association between biochemical recurrence and prostate volume^19^. Evaluating 5,637 patients who underwent radical prostatectomy, a the authors concluded that intermediate-risk patients with lower-volume prostates are more likely to develop biochemical recurrence, which indicates that the relationship between volume and biochemical recurrence may be more complex than it is thought^21^.

In the present work, we set out to test the capacity of a new predictor of biochemical recurrence which is the PSA Density of the lesion. Other authors had already tried to use derivations of the PSA density. However, we did not find studies that used the same criteria of our study to predict biochemical recurrence^22^. The PSA density of the lesion is an estimate of the increase in PSA concentration caused by each gram of the neoplastic lesion. It is an attempt to take into account that there may be small lesions, but with the capacity to cause a relatively high PSA production, or even large neoplastic lesions that do not significantly increase PSA. These would, in theory, be less aggressive, while those would have greater power to cause a clinically unfavorable evolution.

We also tried with this model to correct part of the distortions that occur, for example, when patients who have poorly differentiated lesions and insignificant PSA production have their diagnosis underestimated. The presented results reveal that the proposed new parameter has a statistically significant correlation with the outcome of biochemical recurrence, both in univariate analysis and in multiple logistic regression.

In the context of the immediate postoperative period, we are often faced with the need to decide whether or not the patient would benefit from undergoing any adjuvant treatment either radiotherapy, hormonal block or even chemotherapy^23^. This decision is critical, since all adjuvant therapies have side effects. Our new predictor can help this decision, identifying more aggressive tumors and, therefore, which ones need adjuvant therapies.

Another important point is that the PSA density of the lesion uses data that are normally collected during the diagnosis of PCa. This is important because despite the relatively low rate of prediction (explaining 7% of cases of biochemical recurrence in our cohort), it can help in the prognosis of the disease without bringing any type of harm to the patient, such as a new invasive exam for example.

The usefulness of this new parameter would be to help decide whether to indicate or not adjuvant therapy for patients with a higher risk of biochemical recurrence. The main disadvantage of this work is that it is retrospective analysis. Therefore, mainly prospective studies would be necessary to consolidate the proposed thesis.
